# Physiological and biochemical responses in a cadmium accumulator of traditional Chinese medicine *Ligusticum sinense cv*. Chuanxiong under cadmium condition

**DOI:** 10.1007/s44154-024-00187-5

**Published:** 2024-10-14

**Authors:** Shu-qi Niu, Ting Li, Xiu-wen Bao, Jing Bai, Lin Liu, Si-jing Liu, Wei Qin, Yang Li, Jin-lin Guo

**Affiliations:** 1 College of Medical Technology, Chengdu University of Traditional Chinese Medicine, Chengdu, 610075 China; 2 Chongqing Key Laboratory of Sichuan-Chongqing Co-construction for Diagnosis and Treatment of Infectious Diseases Integrated Traditional Chinese and Western Medicine, Chongqing, China; 3 State Key Laboratory of Southwestern Chinese Medicine Resources, College of Pharmacy, Chengdu University of Traditional Chinese Medicine, Chengdu, 610075 P. R. China; 4 School of Public Health, Chengdu University of Traditional Chinese Medicine, Chengdu, 610075 P. R. China

**Keywords:** Antioxidant system, Cadmium stress, Hormesis effect, *Ligusticum sinense cv.* Chuanxiong, Nitrogen assimilation, Physiological response

## Abstract

*Ligusticum sinense cv.* Chuanxiong (*L. Chuanxiong*), one of the widely used traditional Chinese medicines (TCM), is currently facing the problem of excessive cadmium (Cd) content. This problem has significantly affected the quality and safety of *L. Chuanxiong* and become a vital factor restricting its clinical application and international trade development. Currently, to solve the problem of excessive Cd, it is essential to research the response mechanisms of *L. Chuanxiong* to Cd stress. However, there are few reports on its physiological and biochemical responses under Cd stress. In this study, we conducted the hydroponic experiment under 25 μM Cd stress, based on the Cd content of the genuine producing areas soil. The results showed that 25 μM Cd stress not only had no significant inhibitory effect on the growth of *L. Chuanxiong* seedlings but also significantly increased the chlorophyll a content (11.79%) and root activity (51.82%) compared with that of the control, which might be a hormesis effect. Further results showed that the absorption and assimilation of NH_4_^+^ increased in seedlings under 25 μM Cd stress, which was associated with high photosynthetic pigments. Here, we initially hypothesized and confirmed that Cd exceedance in the root system of *L. Chuanxiong* was due to the thickening of the root cell wall, changes in the content of the cell wall components, and chelation of Cd by GSH. There was an increase in cell wall thickness (57.64 %) and a significant increase in cellulose (25.48%) content of roots under 25 μM Cd stress. In addition, *L. Chuanxiong* reduced oxidative stress caused by 25 μM Cd stress mainly through the GSH/GSSG cycle. Among them, GSH-Px (48.26%) and GR (42.64%) activities were significantly increased, thereby maintaining a high GSH/GSSG ratio. This study preliminarily reveals the response of *L. Chuanxiong* to Cd stress and the mechanism of Cd enrichment*.* It provides a theoretical basis for solving the problem of Cd excessive in *L. Chuanxiong*.

**Graphical Abstract:**

Physiological and biochemical mechanisms of *L. Chuanxiong* seedlings under Cd stress.

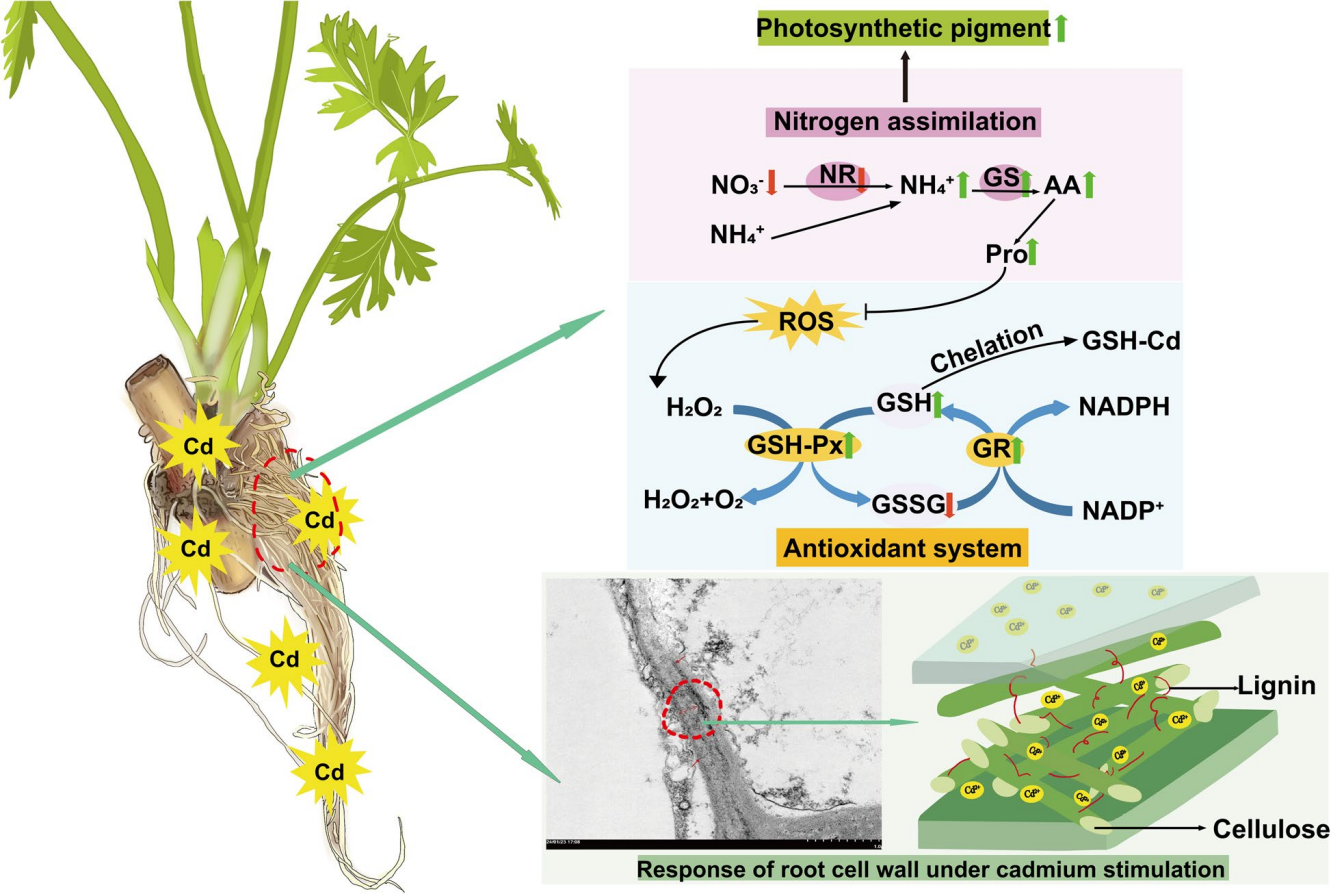

**Supplementary Information:**

The online version contains supplementary material available at 10.1007/s44154-024-00187-5.

## Highlights

1. Under 25 μM Cd, it was the hormesis effect on the growth of *L. Chuanxiong*.

2. Seedlings increased the thickness of root cell wall to chelate and fixate Cd.

3. Seedlings enhanced NH_4_^+^ absorption and assimilation, thereby increasing chlorophyll content.

4. Seedlings mainly reduced oxidative stress caused by Cd stress through the GSH/GSSG cycle.

**Supplementary Information:**

The online version contains supplementary material available at 10.1007/s44154-024-00187-5.

## Introduction

Cadmium (Cd) is one of the major environmental heavy metals (HMs) pollutants, with high mobility, toxicity, and non-degradable characteristics (Yao et al. 2022). For example, Cd is highly mobile in the soil-plant system, and its entry into the food chain would be hazardous to human health (Genchi et al. 2020). Thus, it is also one of the most dangerous pollutants in agriculture.

*Ligusticum sinense cv. *Chuanxiong(*L. Chuanxiong*) is widely applied in traditional Chinese medicine (TCM) for its efficacy in activating blood circulation and removing blood stasis (Chen et al. 2018). In recent years, the problem of Cd excessive in *L. Chuanxiong* medicinal materials has seriously affected its medicinal safety (Xie et al. 2023). The genuine producing area soil of *L. Chuanxiong* usually show a high Cd background with high acidity. In addition, *L. Chuanxiong* is a Cd hyperaccumulator (Yao et al. 2022; Xie et al. 2023). As a result, Chuanxiong (the Rhizome of *L. Chuanxiong*) frequently exceeds the standards (Cd<0.3 mg/kg) of “Chinese Pharmacopoeia (2015 Edition)” and “Green Standards of Medicinal Plants and Preparations for Foreign Trade and Economy”. The problem of Cd excessive in *L. Chuanxiong* urgently needs to be solved. The current traditional methods for solving the problem are ineffective, failing to reduce the Cd content to within the standard range (Xie et al. 2024). Therefore, it is important to understand the physiological and biochemical mechanisms of *L. Chuanxiong* to Cd stress. Although recent studies have explored the response mechanism of *L. Chuanxiong* to different concentrations of Cd stress, they have mainly focused on stress conditions above 5 mg/kg Cd (Zhang et al. 2023; Xie et al. 2024). However, the soil Cd content in genuine producing area of *L. Chuanxiong* is generally around 0.5 mg/kg (Li et al. 2015). Thus, we focused on the effect of low Cd stress on *L. Chuanxiong* seedlings.

The plant root system is the first organ to contact and sense stress and exhibits a range of physiological and biochemical responses. The cell wall is the first barrier that Cd passes through when it comes into contact with the plant root system and enters the cell's interior (Parrotta et al. 2015). Cellulose and lignin, as the main components of the cell wall, play vital roles in the cell wall resistance to Cd stress (Zhong et al. 2018a). Studies have reported that lignin can reduce cell wall permeability and strengthen plant defense against Cd stress by filling up the gaps in the cellulose framework (Kumar et al. 2016). Therefore, the cell wall can reduce the amount of Cd entering the cell to a certain extent, thus mitigating the toxic effects caused by large amounts of Cd entering the organism (Berni et al. 2018).

Upon entering the plant, Cd may lead to metabolic imbalance and the production of a large number of reactive oxygen species (ROS), e.g. superoxide radical (·O_2_^-^), hydroxyl radical (·OH), and hydrogen peroxide (H_2_O_2_) (Gill et al. 2010; Choudhary et al. 2024). Then, it leads to membrane lipid peroxidation, protein oxidation, enzyme inhibition, and nucleic acid damage, ultimately leading to oxidative stress and even cell death (Dobrikova et al. 2021). Thus, oxidative damage is a typical symptom of Cd stress. To reduce the toxic effects of Cd, plants have evolved a series of detoxification strategies. Among them, the antioxidant enzyme system is one of the most critical defense systems (Noctor et al. 2018). The activities of peroxidase (POD), superoxide dismutase (SOD), and glutathione peroxidase (GSH-Px) play a crucial role in the steady-state levels of ROS (Choudhary et al. 2024). The non-enzymatic system consists of small molecules, such as glutathione, vitamins (A, C, and E), and carotenoids (Jaleel et al. 2009). Especially, GSH can protect the plant cellular machinery against ROS-oxidative damage through three potential ways: 1) direct quenching of ROS (Koh et al. 2021); 2) conjugation of HMs and xenobiotic agents to glutathione S-transferase (GST) (Bela et al. 2017); and 3) acting as a precursor for the synthesis of phytochelatins (PCs) (Cao et al. 2017).

As a crucial and abundant mineral nutrient required for plant growth and development, nitrogen (N) is also a key determinant of photosynthetic capacity (Zhong et al. 2018b). Studies on the effects of Cd on N assimilation in plants have shown that Cd inhibits the uptake of inorganic N and the activities of enzymes involved in the N assimilation pathway (Balestrasse et al. 2006). Meanwhile, the damage caused by Cd stress can be effectively mitigated by adding N to improve growth. For example, Zhu et al. (2010) showed that Cd accumulation in *Sedum alfredii* Hance increased with the increase of N levels until it peaked at 16 mM. N addition alleviated high Cd stress and resulted in better growth of poplar plants compared to the control (Zhang et al. 2014). NO plus N conspicuously alleviated Cd-inhibited photosynthetic activity and altered antioxidant enzymes and N assimilation mechanisms in mustard (*Brassica juncea* L.) cv. Giriraj plants (IqbaI et al. 2023). However, there are currently no relevant reports on the relationship between the tolerance and enrichment of Cd in *L. Chuanxiong* and N assimilation.

To subsequently solve the problem of excessive Cd levels in *L. Chuanxiong* rationally, this study aimed to gain insights into the response mechanism of seedlings to 25 μM Cd stress through morphological observation and physiological and biochemical analyses. This study focused on the response of the seedling root system to Cd stress. Firstly, the growth status of seedlings was observed; secondly, the response mechanisms of antioxidant and N assimilation in seedlings were detected; meanwhile, the Cd content and Cd transport capacity in different tissues of seedlings were analyzed. This will lay the foundation for further elucidation of the mechanism of Cd enrichment in *L. Chuanxiong* as well as for addressing the issue of excessive Cd levels.

## Results and discussions

### Effect of Cd stress on seedlings growth of *L. Chuanxiong*

**Fig. 1:**
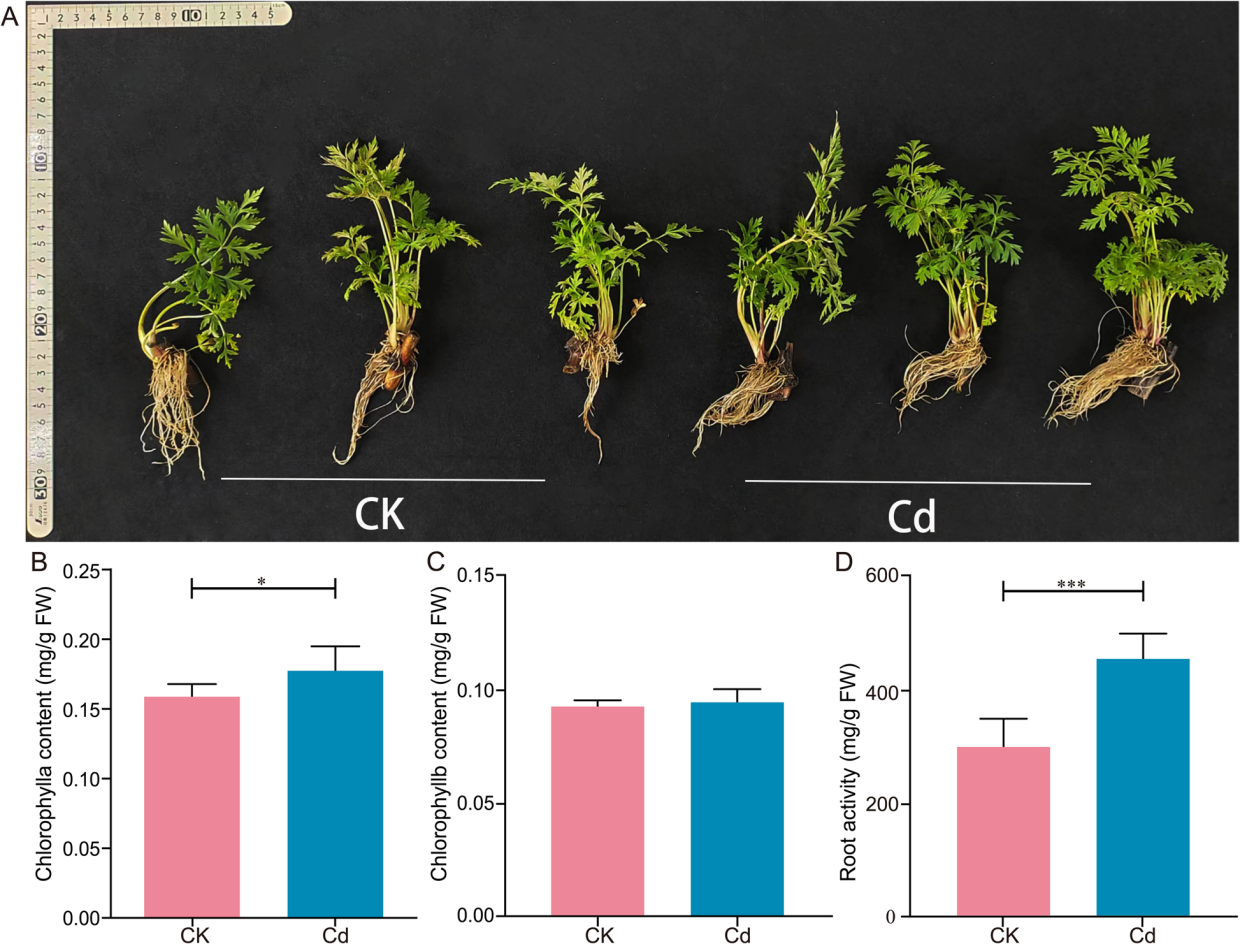
Effect of Cd stress on *L. Chuanxiong* seedling growth phenotype. **A** A representative photograph of seedlings, **B** Chlorophyll a (*n*=4), **C** Chlorophyll b (*n*=4), and **D** Root activity (*n*=9) under CK and 25 μM Cd conditions. Values are means and bars indicate SEs. * and *** indicate significant difference among treatments at *P* < 0.05 and *P* < 0.001 (Tukey-Kramer test)

To determine whether low Cd stress has a stimulatory effect on the growth and development of *L. Chuanxiong* seedlings, we evaluated the effect of 25 μM Cd on seedling growth. There were no significant differences in the growth status of *L. Chuanxiong* between the CK and Cd groups (Fig. 1A, Fig. S1). Notably, the Chlorophyll a (Chl a) content of *L. Chuanxiong* in the Cd group was significantly increased by 11.79% compared with that of CK (Fig. 1B). As the major light-absorbing pigment for plant photosynthesis (Cho et al. 2024), Chlorophyll (Chl) played an important role in plant photosynthesis, specifically in the absorption and utilization of light energy (Yousefi et al. 2018; Zhang et al. 2023). Cd has been shown to disturb photosynthetic activity (Goncharuk et al. 2023), especially Cd stress would reduce the Chl content of plants (Lysenko et al. 2015; Muradoglu et al. 2015), thereby inhibiting photosynthesis and reducing plant growth. In this result, a significant stimulating effect of 25 μM Cd on Chl a content was observed, which might be attributed to hormesis effects. This result was in agreement with previous reports. For example, the total Chl, Chl a, and Chl b content of *Lonicera japonica* Thunb seedlings were all significantly increased by 2.5 and 10 mg·kg^-1^ Cd, but significantly decreased at high Cd concentrations (Jia et al. 2015). Chen et al. (2019) reported that a low dosage of Cd enhanced the synthesis of Chl a and Chl b in the plants. It was also reported that *Perilla frutescens*, a Cd accumulator, showed a slight increase in Chl a and Chl b contents with prolonged treatment at low Cd levels (Xiao et al. 2020). Cd was mainly absorbed into plants through the root system. Root activity was an important indicator of root absorption capacity (Xiao et al. 2020). In the present study, the root activity of *L. Chuanxiong* in the Cd group was significantly increased by 51.82 % compared with that of the CK group (Fig, 1D). This result reflected that the root system had a high absorption capacity under 25 μM Cd.

As a hyperaccumulator plant, recent reports have focused on exploring the harmful effects of high Cd stress on *L*. *Chuanxiong*. For example, Zhang et al. (2023) revealed that Cd stress (5, 10, 20, and 40 mg·kg^-1^) inhibited biomass accumulation and root development through physiological and transcriptomic analyses. Xie et al. (2023) reported that ferulic acid content, an important indicator of *L. Chuanxiong* quality, was greatly influenced by Cd stress (5 and 10 mg·kg^-1^). Notably, some plants have toxic excitation effects, specifically manifested as plants can maintain or even improve their performance under Cd exposure, although the Cd accumulation in plant tissues (Carvalho et al. 2020). Therefore, these results further supplement the impact of low Cd stress on the growth of *L. Chuanxiong* seedlings. In sum, high doses of Cd had a toxic effect on *L. Chuanxiong*, while low doses of Cd stimulated growth.

### Effect of Cd stress on root cells of *L. Chuanxiong*

**Fig. 2:**
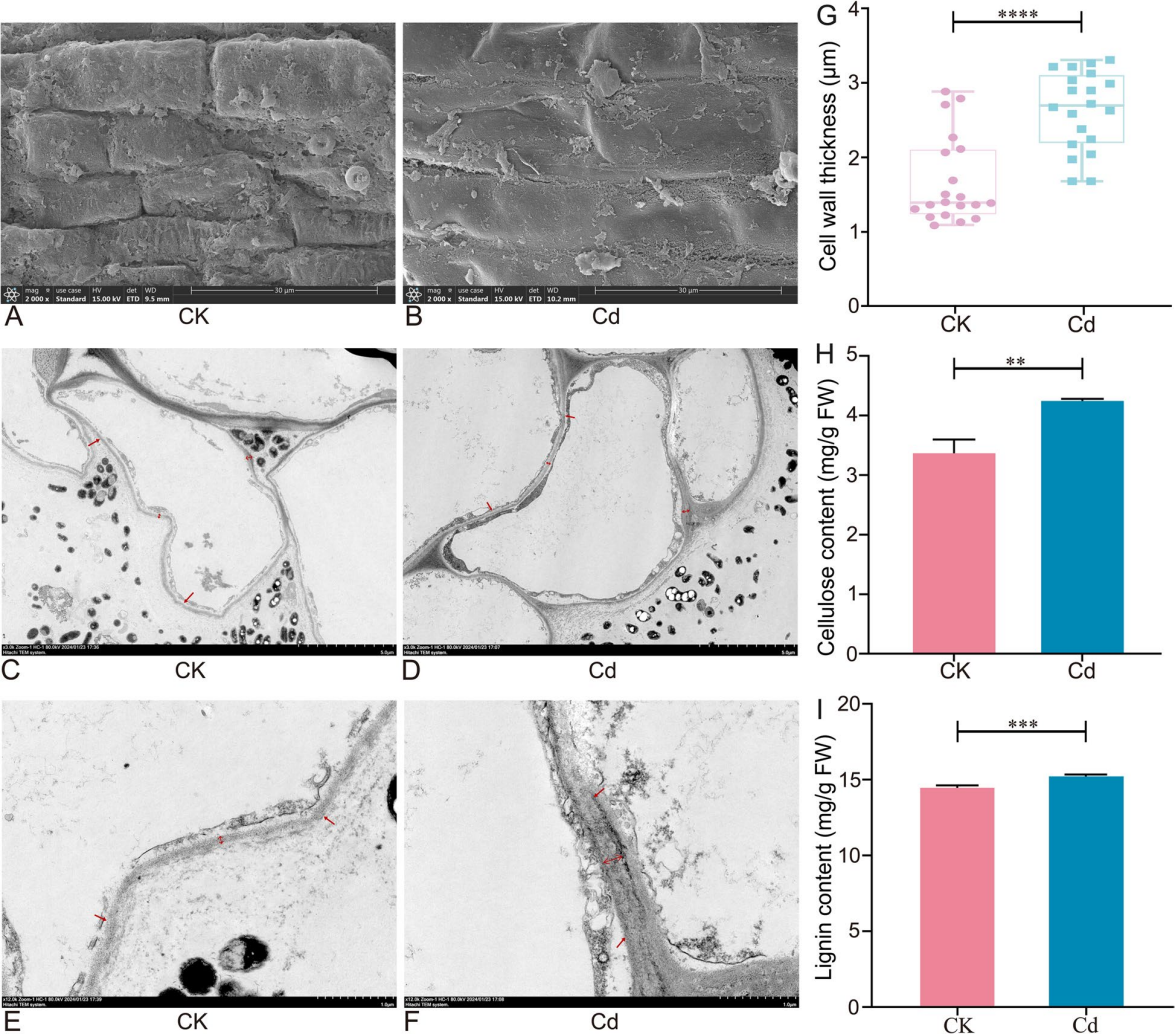
Effect of Cd stress on root cells of *L. Chuanxiong* seedling. **A** and **B** SEM, **C**-**F** TEM. **G** Cell wall thickness, values are means and bars indicate SEs (*n* = 20). **H** Cellulose, and **I** Lignin content of seedlings under CK and 25 μM Cd conditions. Values are means and bars indicate SEs (*n* = 3). **, ***, and **** indicate significant difference among treatments at* P* < 0.01, *P* < 0.001, *P* < 0.0001, respectively (Tukey-Kramer test)

To analyze the effect of Cd on the root system of seedlings, we performed microscopic observations. As shown in Fig. 2A-G, there was cell wall thickeness in the roots significantly increased (57.64%) under 25 μM Cd, compared to that under CK condition. This was also in line with previous reports (Zhang et al. 2023; Xie et al. 2024). The above results might be due to the influence of Cd on the cell wall, leading to changes in its biosynthesis. Therefore, we further determined the contents of the main components of the cell wall. Compared with the CK group, cellulose and lignin contents were significantly increased, with the cellulose content by 25.48% (Fig. 2H). As the key components of the cell wall, the increase in its content can further indicate the thickening of the root cell wall. The cell wall was the first structure of plant cells to come in contact with HMs (Parrotta et al. 2015), which could act as a barrier to prevent HM ions from entering the plant cytoplasm (Khaliq et al. 2019). As reported, the cell wall could sequester HMs due to its composition that conferred the ability to bind HMs via functional groups (Parrotta et al. 2015). Combined with previous reports, Cd stored in the cell wall of *L. Chuanxiong* had little translocation capacity (Xie et al. 2024). Additionally, Cd subcellular distribution analysis demonstrated that Cd was mainly stored in the root of *L. Chuanxiong* (10-fold more than in the leaf), whose Cd content was cytoderm > cytoplasm > organelle in tissues (Xie et al. 2024). These are also in agreement with our results, suggesting that the thickening of the root cell wall and changes in its composition are mechanisms of resistance to Cd and one of the reasons for the Cd exceedance in the medicinal part.

### Effect of Cd stress on Cd content and transport of *L. Chuanxiong* seedlings

**Fig. 3:**
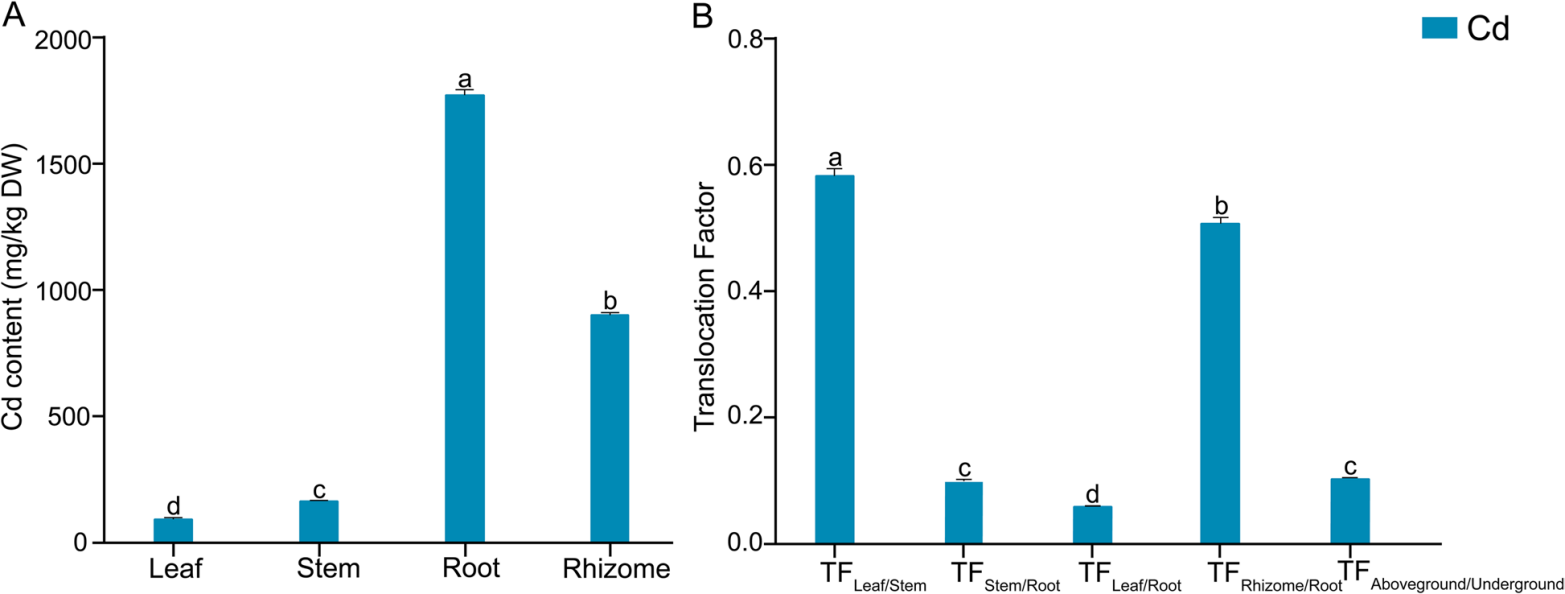
Effect of Cd stress on Cd content and TF of *L. Chuanxiong* seedling. **A** Cd content and **B** TF of different tissues of seedlings under 25 μM Cd conditions. Values are means and bars indicate SEs (*n* = 3). Different letters indicate significant difference among treatments at *P* < 0.05 (ANOVA and Duncan’s multiple range test)

As shown in Fig. 3A and Table S1, the Cd content in roots, stems, and leaves reached by 1782.50 mg·kg^-1^, 173.42 mg·kg^-1^, and 101.85 mg·kg^-1^ under 25 μM Cd, respectively. Meanwhile, the Cd content in the CK group wasn’t obtained, because of being below the detection limit of the device. There were differences in Cd content among different parts of plants, which were closely related to their characteristics and ability to accumulate HMs (Yao et al. 2022). Similarly, most hyperaccumulator plants stored Cd mainly in the roots. Consistent with previous reports, the Cd content in the roots of *L. Chuanxiong* was significantly higher than that in the other parts (Zhang et al. 2023). Translocation factors (TFs) could indicate the ability of Cd to transport from one tissue to the next (Li et al. 2022). As shown in Fig. 3B, the TF_leaf/stem_ (0.59) and TF_rhizome/root_ (0.51) further confirmed *L. Chuanxiong* had a high capacity to transport Cd, especially from stem to leaf and root to rhizome. Meanwhile, the low TF_aboveground/underground_ (0.10) indicated that Cd accumulation in *L. Chuanxiong* seedlings was mainly in the underground. All these data confirmed that the low Cd transport capacity in the underground part of *L. Chuanxiong* was the main reason for the exceedance of Cd in medicinal parts.

### Effect of Cd stress on nitrogen assimilation of *L. Chuanxiong* seedlings

**Fig. 4:**
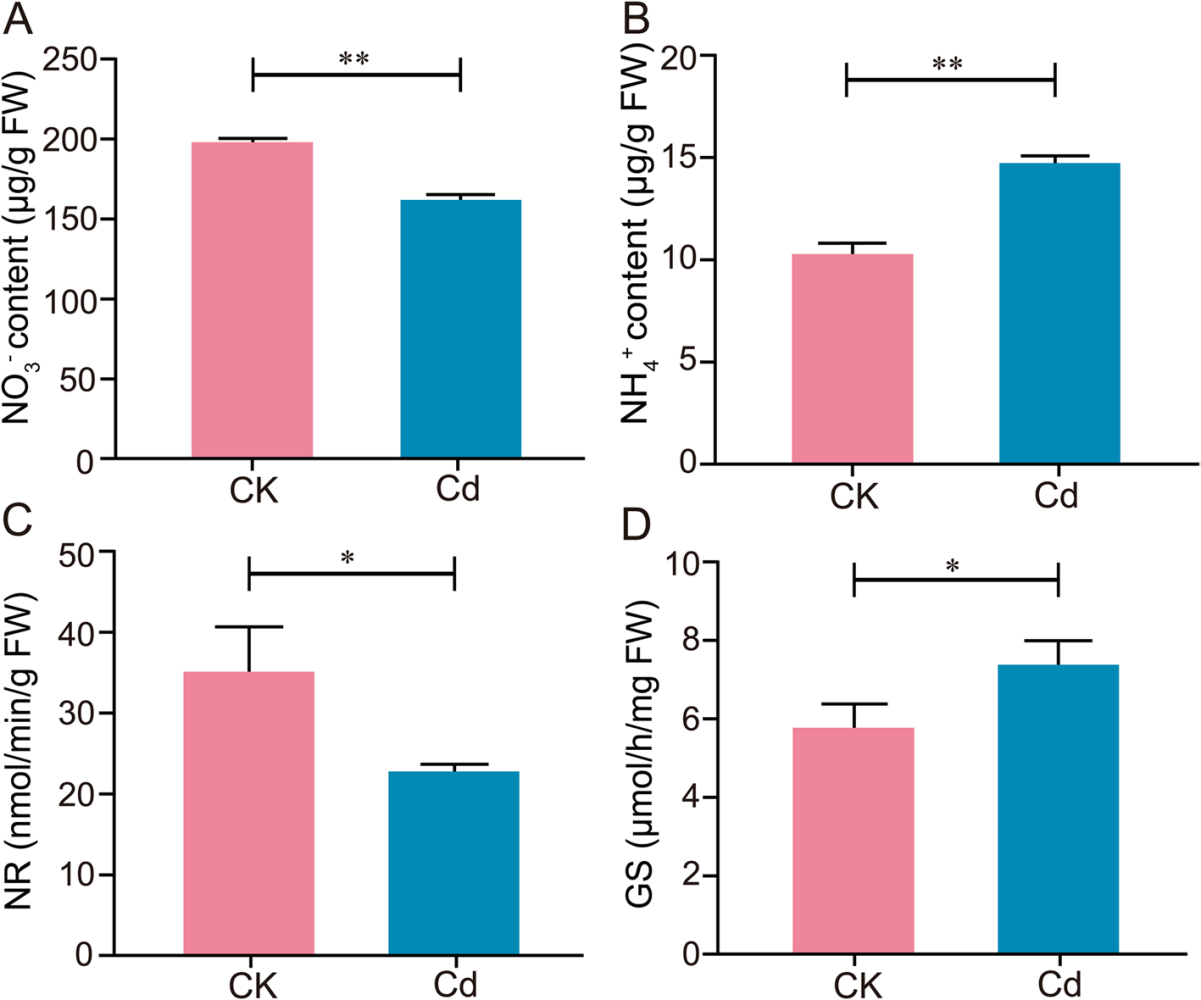
Effect of Cd stress on inorganic N content and N assimilation enzyme activity of *L. Chuanxiong* seedling. **A** NO_3_^-^, **B** NH_4_^+^, **C** NR and **D** GS activity of seedlings under CK and 25 μM Cd conditions. Values are means and bars indicate SEs (*n* = 3). * and ** indicate significant difference among treatments at *P* < 0.05, *P* < 0.01, respectively (Tukey-Kramer test)

N was essential for plant growth and development, and resistance against biotic and abiotic stresses (Kishorekumar et al. 2020). Cd could affect N assimilation (Zaid et al. 2020), such as hindering the activity of NR and NiR (Hussain et al. 2020), and the activity of enzymes involved in NH_4_^+^ assimilation (Goncharuk et al. 2023). As reported, N assimilation contributed to photosynthetic pigment biosynthesis (Wang et al. 2023). Considering the correlation between N and photosynthetic pigment content, we further determined the N-related indicators in conjunction with the experimental results of the increase in Chl a content of *L. Chuanxiong* under Cd stress. As shown in Fig. 4A and B, Cd induced higher NH_4_^+^ content in the roots of the Cd group than that of the CK group, while NO_3_^-^ content was the opposite. Moreover, we also observed that the pH of the hydroponic solution under Cd stress was significantly lower than that of the CK group, especially 4 days after treatment (Fig. S2). Thus, we speculated that *L. Chuanxiong* biased toward absorbing NH_4_^+^. Correspondingly, the activity of GS in the Cd group was significantly increased by 28.55% and the NR activity was significantly decreased by 34.90% relative to the CK group (Fig. 3C and D). NR mediated the initiation of inorganic N utilization, and GS converted inorganic N to organic N, playing a crucial role in N assimilation (Kishorekumar et al. 2020; Cho et al. 2024). Notably, the reduction of 1 mol NO_3_^−^ to NH_4_^+^ required 8-12 mol ATP (Zhong et al. 2018b). Therefore, it speculated that the reduction of NO_3_^-^ metabolism by *L. Chuanxiong* seedlings was an energy-saving behavior under Cd stress. In all, it suggested that *L. Chuanxiong* might enhance the ability of NH_4_^+^ assimilation under low Cd stress.

**Fig. 5:**
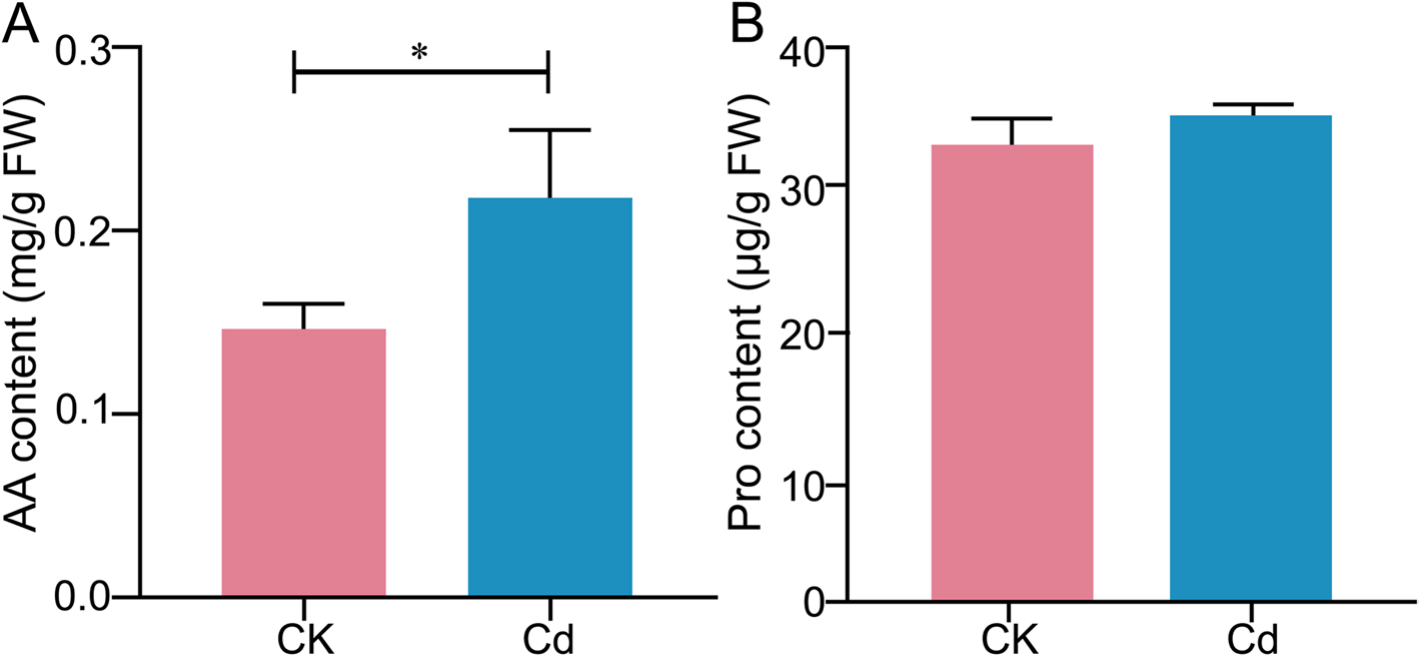
Effect of Cd stress on AA and Pro content of *L. Chuanxiong* seedling. **A** AA and **B** Pro content of seedlings under CK and 25 μM Cd conditions. Values are means and bars indicate SEs (*n* = 3). * indicates significant difference among treatments at *P* < 0.05 (Tukey-Kramer test)

Ultimately, the amino acid (AA) content significantly increased, with an increasing trend in Proline (Pro) content (Fig. 5). Therefore, we speculated that *L. Chuanxiong* seedlings increased NH_4_^+^ assimilation, which provided N backbone for Chl, and thus enhanced their photosynthetic capacity, thereby promoting growth under Cd stress. The physiological response of *L. Chuanxiong* plants to low Cd-mediated NH_4_^+^ assimilation can be used in augmenting growth under Cd stress.

### Effect of Cd stress on antioxidant system of *L. Chuanxiong* seedlings

**Fig. 6:**
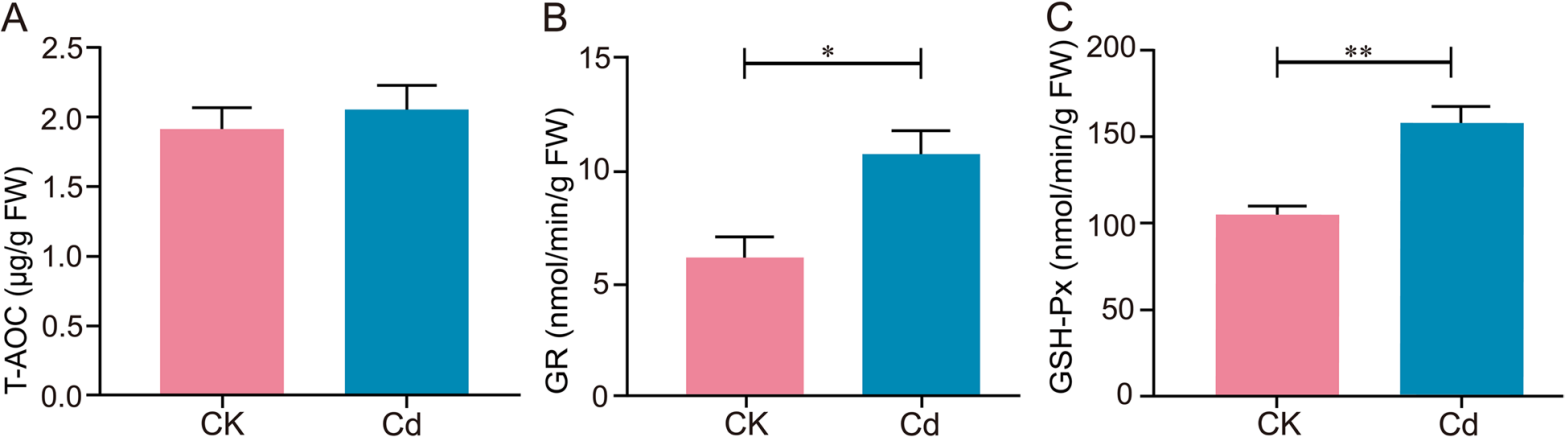
Effect of Cd stress on antioxidase activity of *L. Chuanxiong* seedling. **A** T-AOC, **B** GR, and **C** GSH-Px of seedlings under CK and 25 μM Cd conditions. Values are means and bars indicate SEs (*n* = 3). * and ** indicates significant difference among treatments at *P* < 0.05 and *P* < 0.01 (Tukey-Kramer test)

To alleviate the oxidative damage initiated by Cd stress, plants usually activate antioxidant defenses to maintain cellular redox homeostasis (Zhu et al. 2021). In this study, there was no significant difference in the MDA content of seedling roots (Fig. S3), indicating the roots weren't damaged by 25 μM Cd stress and that *L. Chuanxiong* had the ability of Cd tolerance. Then, we further investigated the antioxidant defense mechanism, focusing on the antioxidant enzyme scavenging system. Notably, as shown in Fig. 6, GSH-Px (48.26%) and GR (42.64%) activities significantly enhanced under Cd stress relative to the CK group. Meanwhile, other antioxidant enzymes showed an increasing trend, but there was no significant difference (data not shown). As reported, enhanced activities of antioxidative enzymes could help detoxify ROS and reduce oxidative damage, ultimately improving plant stress resistance (Mishra et al. 2023; Kumar et al. 2024). For example, plants could enhance the activity of antioxidative enzymes, thereby protecting the photosynthetic electron transport from Cd oxidative damage by maintaining the NADP/NADPH ratio (Qamer et al. 2021). Recently, studies on the relationship between *L. Chuanxiong* and Cd stress had shown that under a certain concentration range of Cd stress (5-40 mg·kg^-1^ Cd), the activities of various enzymes such as SOD, POD, and CAT in the antioxidant system significantly increased, indicating that the antioxidant system of *L. Chuanxiong* strengthened with increasing Cd concentration (Xie et al. 2023; Zhang et al. 2023). As a result, *L. Chuanxiong* reduced or even eliminated the impact of stress on its growth by precisely regulating its antioxidant system, especially GSH-Px and GR enzyme activities.

**Fig. 7:**
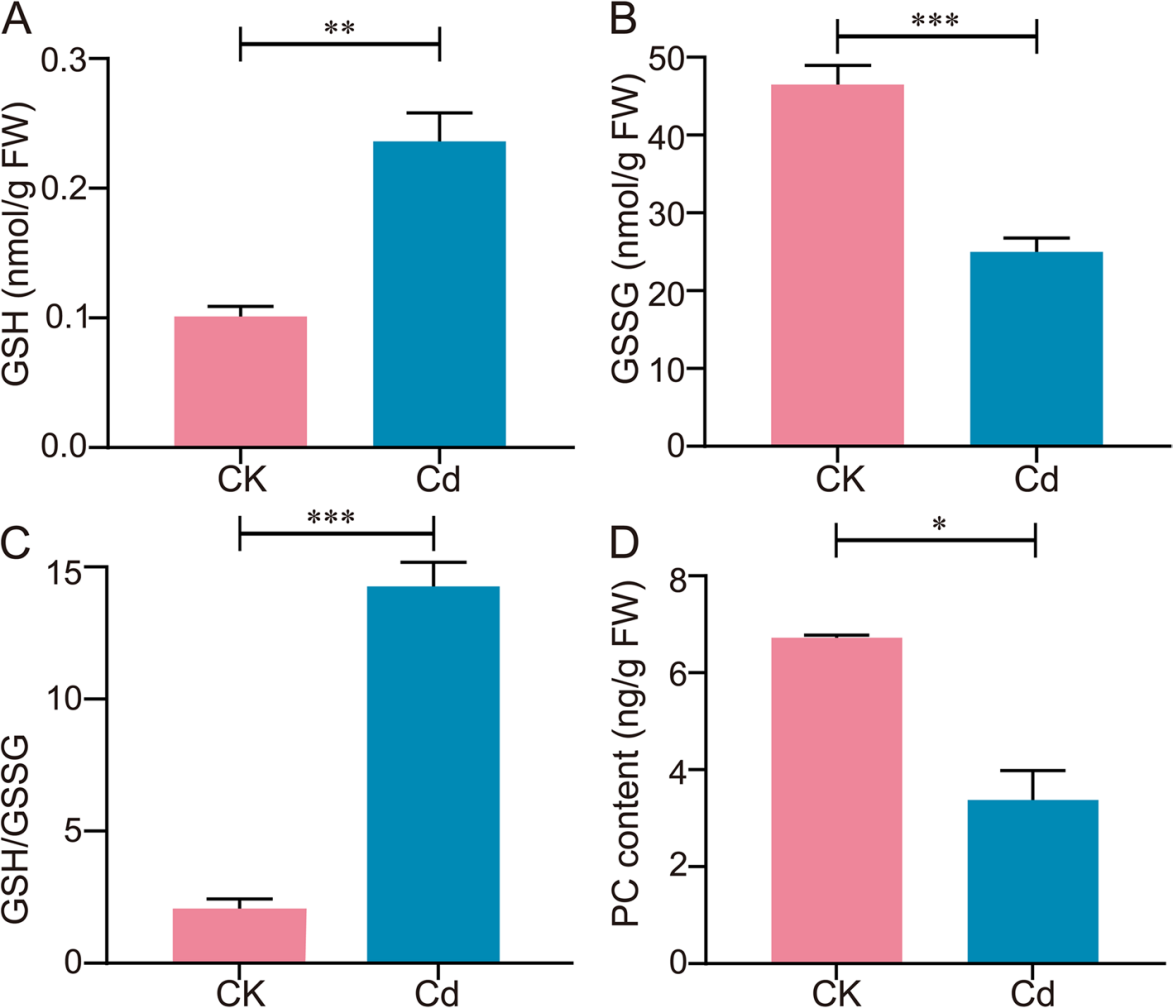
Effect of Cd stress on non-enzymatic system and PC content of *L. Chuanxiong* seedling. **A** GSH, **B** GSSG, **C** GSH/GSSG and **D** PCs content of seedlings under CK and 25 μM Cd conditions. Values are means and bars indicate SEs (*n* = 3). *, **, and *** indicate significant difference among treatments at *P* < 0.05, *P* < 0.01, and *P* < 0.001, respectively (Tukey-Kramer test)

Additionally, several non-enzymatic mechanisms were also involved in this defense, including antioxidants such as Pro and GSH. ﻿﻿In this study, Cd-induced higher Pro and GSH contents in the root under Cd stress than that of the CK group (Fig. 5B, Fig. 7A), which could explain why the MDA content had no significant changes between CK and Cd groups. As a low-molecular-weight AA, Pro is an essential antioxidant that functions as a key ROS scavenger, maintaining the cellular redox of plants (Koh et al. 2021). In addition, Pro was also known as an osmoprotectant, acting as a molecular chaperone to protect the integrity of protein and membrane (Niu et al. 2022). GSH and its metabolites play an important role in the absorption, transportation, accumulation, and detoxification of Cd in plants (Genchi et al. 2020). GSH can act as a reducing agent to remove the excess H_2_O_2 _induced by abiotic stress,  while being oxidized to oxidized glutathione (GSSG) by GSH-Px (Koh et al. 2021; Kumar et al. 2024), which in turn is reduced to GSH by GR (Cao et al 2017; Koh et al. 2021), further maintaining the GSH/GSSG homeostasis. Under Cd stress, the GSH content of *L. Chuanxiong* significantly increased, while the GSSG content markedly decreased, maintaining a high GSH/GSSG level (Fig. 7). This indicated that the transformation cycle between GSH and GSSG facilitates the removal of excess ROS accumulation. The above results again suggested that *L. Chuanxiong* seedlings regulated the antioxidant system to cope with Cd stress, especially by maintaining a high GSH/GSSG ratio. Meanwhile, a high GSH/GSSG ratio was also beneficial for protein synthesis (Dorion et al. 2021). This was also consistent with the previous result of increased AA content under Cd stress. As reported, Cd stress significantly affected the synthesis of GSH in plants, exhibiting a "low promotion and high inhibition" effect (Fan et al. 2022; Li et al. 2023). Another report suggested that exogenous GSH could improve the photosynthetic performance of plants, with a significant increase in photosynthetic pigment levels such as Chl a and Chl b in GSH-treated wheat seedlings subjected to Cd stress (Li et al. 2021). Therefore, in this study, the high GSH content of *L. Chuanxiong* seedlings under Cd stress may be important in regulating photosynthetic activity and promoting growth.

GSH could also directly bind with Cd to form chelating substances, or GSH could further form PCs to chelate Cd (Li et al. 2022). Usually, Cd in the soil was easily and quickly absorbed by plant roots (Khaliq et al. 2019), which were important parts of Cd absorption and accumulation. Specifically, after the root absorbed Cd, it was transported to the aboveground part of the plant through the xylem. However, the participation of GSH and PCs could promote the binding of Cd in the plant roots, causing it to remain in the plant roots and avoiding its transfer to the aboveground part (Seth et al. 2012). It was proved that the supplementation of GSH improved the vacuole compartmentalization and was crucial for the detoxification of Cd (Fang et al. 2020). In particular, two sulphydryl (SH) groups from GSH and/or PCs could complex one Cd^2+^ (Genchi et al. 2020). Both our study and related research have shown that the Cd content in the roots of *L. Chuanxiong* was higher than in other tissues (Xie et al. 2023; Zhang et al. 2023), suggesting that Cd could chelate into the roots of *L. Chuanxiong*. To determine how the higher content of GSH exerted chelating effects, we further measured the PCs content of *L. Chuanxiong*. As shown in Fig. 7D, the PCs content was lower in seedlings under Cd stress relative to the CK group, suggesting the direct chelation of GSH with Cd under low Cd stress. There were also relevant reports in other plants. For example, GSH could complex all Cd present in Cd-treated leaves of tobacco plants (Vögeli-Lange and Wagner 1996). The GSH had a vital role in ameliorating lipid peroxidation and alleviating Cd-induced toxicity in maize plants (Wang et al. 2021). Additionally, previous studies have reported that the PCs content in *L. Chuanxiong* seedlings significantly increased under high Cd stress, leading to responsive chelation (Xie et al. 2023). Based on the results of this experiment, we further speculated that GSH not only exerted antioxidant effects but also directly chelated Cd under low Cd stress.

## Conclusion

This study investigated the effects of Cd stress on the growth and development of *L. Chuanxiong* seedlings and proposed insights into Cd accumulation and tolerance mechanisms in roots. Under 25 μM Cd stress, it didn’t affect the growth of seedlings but produced a hormesis effect. Specifically, it promoted NH_4_^+^ assimilation, thereby increasing the total AA and Chl a content, and also enhanced root activity. Consistently, *L. Chuanxiong* regulated its antioxidant system by maintaining a high level of GSH/GSSG and increasing GSH-Px activity to reduce or eliminate ROS bursts caused by Cd stress. The changes in the content of cell wall components and the thickening of the root cell wall under Cd stress may be one of the reasons for the Cd exceedance in the underground part. The high content of GSH may also play a role in chelating Cd, which will help us to elucidate the enrichment mechanism of Cd in *L. Chuanxiong* more deeply in the future. This study can also provide a basis for solving the problem of excessive Cd in *L. Chuanxiong*.

## Materials and methods

### Plant growth and Cd treatment

*L. Chuanxiong* seedlings were cultivated at the Pharmacy College, Chengdu University of Traditional Chinese Medicine, Chengdu, Sichuan Province, China (30°41′N, 104°48′E). After 15 days, the seedlings with similar growth conditions were selected, and each seedling was washed with distilled water. Then, the seedlings were transferred to a nutrient solution filled with 800 mL of Hoagland hydroponic (including 10 μM H_2_BO_3_, 0.5 μM MnSO_4_, 5 μM ZnSO_4_, 0.2 μM CuSO_4_, 0.01 μM (NH_4_)_6_MO_7_O_24_, 20 μM C_10_H_12_FeN_2_NaO_8_·3H_2_O, 0.05 mM CaCl_2_, 0.5 mM MgSO_4_, 0.1 mM KH_2_PO_4_, 0.7 mM K_2_SO_4_, 0.1 mM KNO_3_, 1.95 mM Ca(NO_3_)_2_) being replaced every 2 days. After 6 days, the seedlings were transferred to a hydroponic solution containing 25 μM CdCl_2_ and without Cd, and cultured for another 6 days. The physiological and biochemical parameters were measured.

### The measurement of plant growth physiological parameter

After Cd stress treatment, samples of *L. Chuanxiong* seedlings were collected from each group. The seedling samples were washed several times with distilled water. The height and fresh weight of seedlings were measured, and then the dry weight of seedlings was obtained after drying the samples in an oven at 60 °C.

The chlorophyll a (Chl a) content of leaves was determined by the acetone-ethanol method (Niu et al 2016). Briefly, fresh leaf samples (0.1 g) of *L. Chuanxiong* were placed in a 15 mL centrifuge tube, and immediately added 80% acetone (5 mL) and 95% ethanol (5 mL) in the dark for 48 h. Absorbance was measured by a UV spectrophotometer (UV-721G, Instrumental Analytical Instruments Co., Ltd., Shanghai, China) at 663 and 645 nm, respectively.

The root activity of seedlings was determined by TTC (triphenyltetrazolium chloride) method (He et al 2018). Briefly, the fresh root tips (0.1 g) were put into the tube with the 10 mL mix of 0.4 % TTC and phosphate buffer (PBS) in equal volume, and keep them warm for 3 h at 37 °C in the dark. Then add 2 mL of 1 mol·L^-1^ sulfuric acid to stop the reaction (add sulfuric acid first and then root as a blank test, other operations are the same as above). Take out the root and absorb the water with filter paper, put it into a test tube with 10 mL of methanol, seal the nozzle with parafilm and put it in a dark place for 3 h, wait for the root to retreat to white at the wavelength of 485 nm, and use the blank test as a control to measure its absorbance value (UV-721G, Instrumental Analytical Instruments Co., Ltd., Shanghai, China).

### Microscopic observation of roots

Root samples of *L. Chuanxiong* seedlings were collected and fixed in a 3% glutaraldehyde fixative solution for more than 24 h. For the scanning electron microscope (SEM) obseravtion, the fixed samples were rinsed, dehydrated with gradient alcohol, dried at the critical point, and selected for observation under an electron microscope with a suitable viewing surface (Apreo S, Thermo Fisher Technology Co., Ltd., Massachusetts, America). For the transmission electron microscope (TEM) obseravtion, root sample pieces of approximately take 1 mm^3^ and rinse with PBS 3 times for 15 min each time. Then, the samples were fixed with 1% osmium acid (in 0.1 M PBS) for 2 h, rinsing steps as above. After the gradient elution of alcohol, pure acetone was added for two dehydration treatments of 15 min each. Acetone and embedding agent were mixed and infiltrated in equal proportions for 3 h, and then mixed and infiltrated at 2:1 for overnight embedding treatment. After polymerization, sectioning, and staining, the samples were observed under a TEM and images were collected for analysis (HT7800, Hitachi Co., Ltd., Tokyo, Japan).

### Determination of cell wall components

The lignin content was determined according to the method of Kong et al. (2018). The cellulose content was determined according to the method of Broxterman et al. (2018) with appropriate adjustments. A fresh sample of 0.1 g was extracted with 80% ethanol and pure acetone, and the precipitate was retained. The precipitate was treated with the sulfite-NaOH method to obtain supernatant and absorbance was measured at 620 nm.

### The measurement of inorganic N content

For determination of inorganic N content of seedlings, the nitrate nitrogen (NO_3_^‐^-N) and ammonium nitrogen (NH_4_^+^-N) were measured using the reagent kits (Suzhou Michy Biomedical Technology Co., Ltd, Suzhou, China).

### The measurement of N assimilation parameters

Root samples of seedlings were weighed, and the activity of enzymes related were determined. Glutamine synthetase (GS) activity was determined according to the methods of Zhong et al (2018b). Nitrate reductase (NR) activity was measured following Park et al (2011). Amino acid (AA) content was determined by the ninhydrin reaction following Friedman et al. (2004).

The free proline (Pro) content was determined by the sulfosalicylic acid method (He et al. 2018). Briefly, proline was extracted from 0.1-g samples into 3% sulfosalicylic acid (10 mL), then filtered, the absorbance was determined at 520 nm.

### The measurement of antioxidant system parameters

Malondialdehyde (MDA) content was determined following Niu et al (2022). Briefly, the roots sample (0.1 g) was macerated in 5 mL trichloroacetic acid (0.1%), and the homogenate was centrifuged. Then, 1 mL of the supernatant was mixed with 4 mL of 20% TBA (containing 0.5% thiobarbituric acid). Finally, the obtained mixture was centrifuged, and the absorbance of the supernatant was determined at 532 and 600 nm.

For antioxidant enzyme activity, GSH-P_X_ activity was measured following Gajewska et al (2007). The total antioxidant capacity (T-AOC) was determined by using the reagent kit. The reduced glutathione (GSH) and oxidized glutathione (GSSG) levels were estimated by using the reagent kits. For the GSH assay, GSH was oxidated by the sulfhydryl reagent 5,5’-dithiobis (2-nitrobenzoic acid) (DTNB) to form the yellow derivative 5’-thio-2-nitrobenzoic acid (TNB), and measurable at 412 nm. For the GSSG assay, Glutathione reductase (GR) could catalyze the oxidation-reduction reaction between GSSG and NADPH, reducing GSSG to GSH. GSH reacted with DTNB, which reflected GSSG content. GR activity was determined by using the reagent kit. Briefly, GR can catalyze NADPH to reduce GSSG and regenerate GSH, while NADPH dehydrogenates to produce NADP^+^. The rate of NADPH dehydrogenation was determined by measuring the rate of decrease in absorbance at 340 nm to calculate GR activity. The phytochelatins (PCs) level was measured using a dual-antibody sandwich enzyme-linked immunosorbent assay. The above reagent kits were from Suzhou Michy Biomedical Technology Co., Ltd, Suzhou, China.

### The measurement of *L. Chuanxiong* Cd content

The dried samples (0.1 g) of roots, stems, and leaves were put into the polytetrafluoroethylene digestion inner tank, and 5 mL of nitric acid was added to soak overnight. Then, put it into the constant temperature drying oven at 80 °C for 1-2 h, keep it at 120 °C for 1-2 h, and rise to 160 °C for 4 h. Next, cool it naturally to room temperature in the constant temperature drying oven. Finally, dilute the digestive solution to a volumetric flask. The inductively coupled plasma mass spectrometry (ICP-MS, iCAP RQ Thermo Fisher Technology Co., Ltd., Massachusetts, USA) was used to determine the Cd content in *L. Chuanxiong* samples.

For the translocation factor (TF) was calculated using the following equations by Zhang et al. (2021): TF = Cd _aboveground_/Cd _underground,_ where Cd _aboveground_ and Cd _underground_ were the Cd concentration in different parts, respectively.

### Statistical analysis

All experiments have three replicates. The bars with different letters are significantly different (ANOVA, *P* < 0.05) according to Duncan’s multiple-range test (version 17.0, SPSS Inc., Chicago, IL, USA). The bars with asterisks are significantly different as determined by Tukey-Kramer test (*P < 0.05, **P < 0.01, ***P < 0.001, ****P < 0.0001). GraphPad Prism version 6.0 software was used for plotting.

## Supplementary Information

Supplementary Material 1.

Supplementary Material 2.

## Data Availability

Data and materials will be made available on request.

## Abbreviations

AA: Amino acid
Cd: Cadmium
*L. Chuanxiong*: *Ligusticum sinense cv.* Chuanxiong
GR: Glutathione reductase
GS: Glutamine synthetase
GSH: Reduced Glutathione
GSH-Px: Glutathione peroxidase
GSSG: Oxidized Glutathione
GST: Glutathione S-transferase
SEM: Scanning Electron Microscope
HM: Heavy metal
MDA: Malondialdehyde
N: Nitrogen
NR: Nitrate reductase
PCs: Phytochelatins
POD: Peroxidase
Pro: Proline
ROS: Reactive oxygen species
SOD: Superoxide dismutase
T-AOC: Total antioxidant capacity
TBA: Thiobarbituric acid
TCM: Traditional Chinese medicines
TEM: Transmission Electron Microscope
TF: Translocation factor
