# Supplementary material for: Physiological and biochemical responses in a cadmium accumulator of traditional Chinese medicine Ligusticum sinense cv. Chuanxiong under cadmium condition
Source: Stress Biol. 2024 Oct 14;4(1):44. doi: 10.1007/s44154-024-00187-5 (PMC11473752; doi:10.1007/s44154-024-00187-5)
Supplement: Supplementary file 1 — Supplementary Material 1. [file 44154_2024_187_MOESM1_ESM.doc]

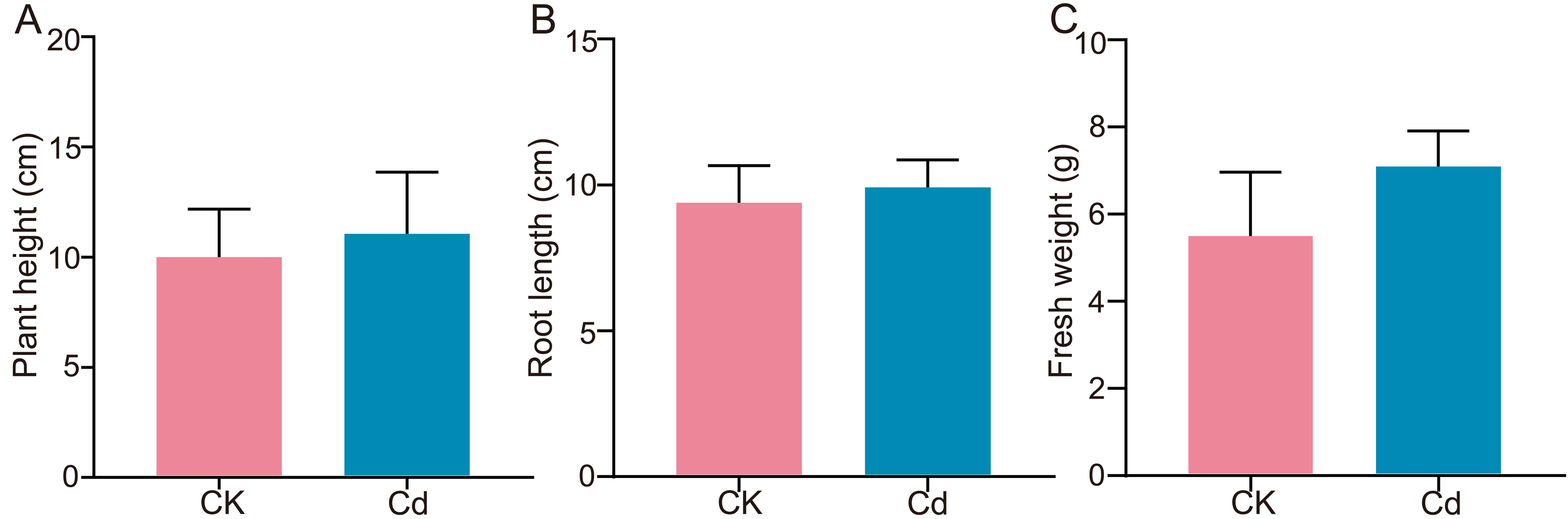
Figure S1. Effect of Cd on *L. Chuanxiong* seedling of plant height (A), root length (B), and fresh weight (C). Values are means and bars indicate SEs (n = 6).


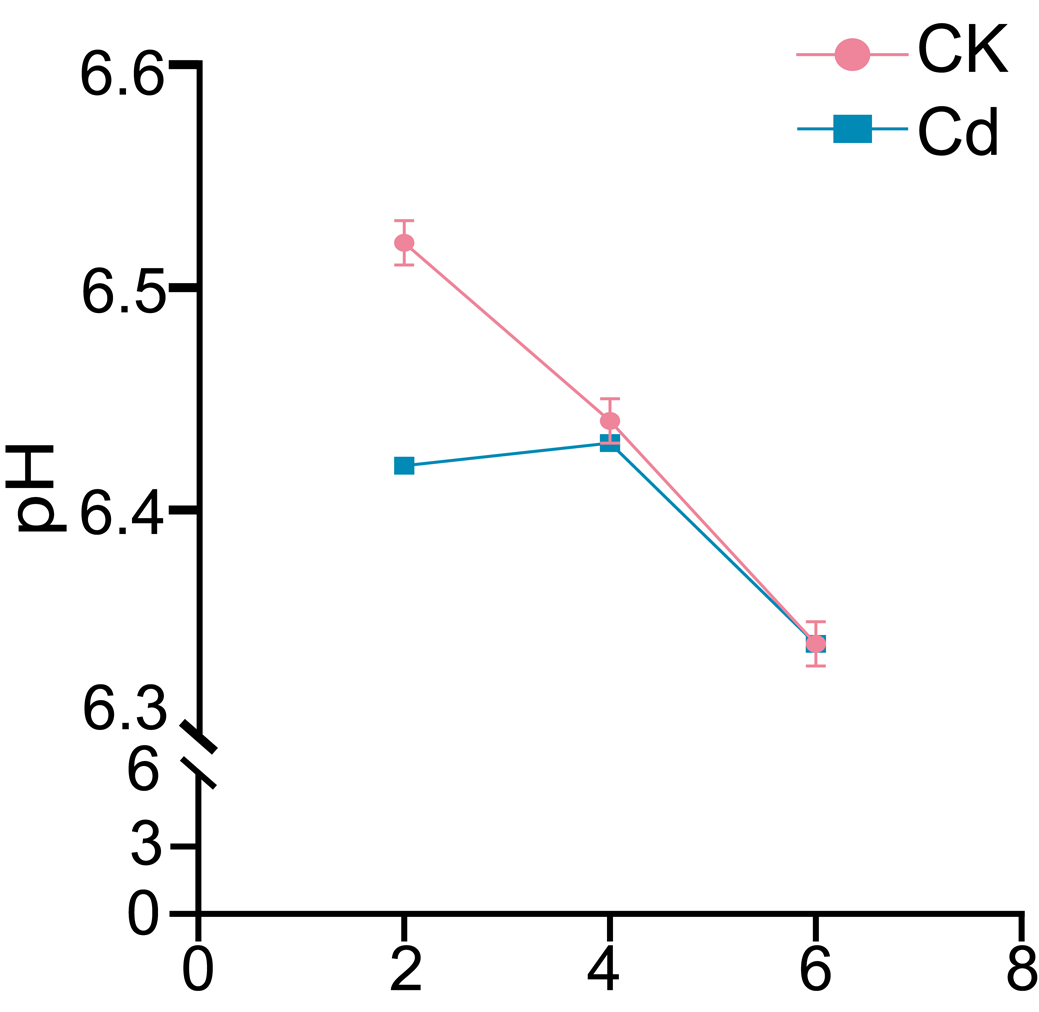
Figure S2. Effect of Cd on pH of hydroponic solution. Values are means and bars indicate SEs (n = 3).


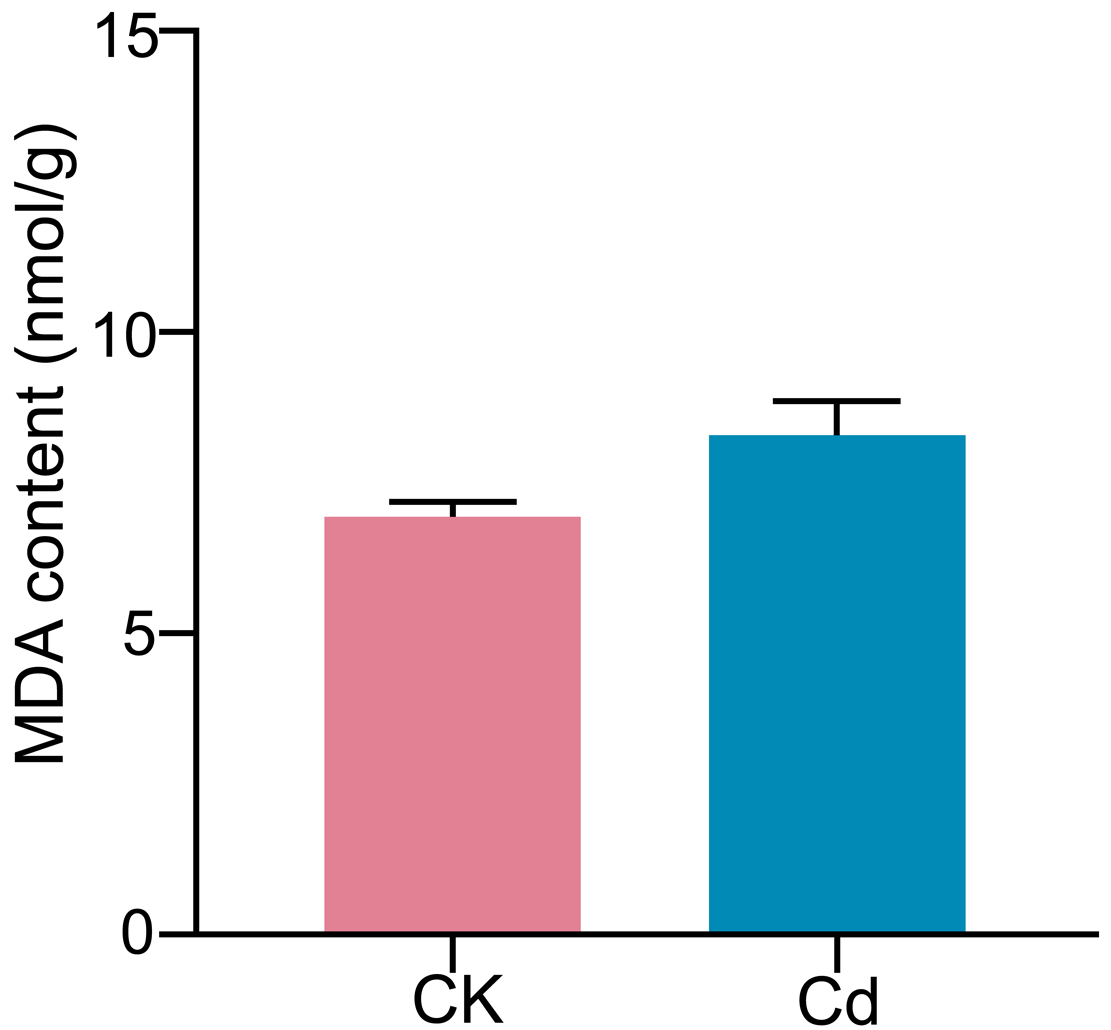
Figure S3. Effect of Cd on MDA content of *L. Chuanxiong* seedlings. Values are means and bars indicate SEs (n = 3).
