# Supplementary material for: Physiological and biochemical responses in a cadmium accumulator of traditional Chinese medicine Ligusticum sinense cv. Chuanxiong under cadmium condition
Source: Stress Biol. 2024 Oct 14;4(1):44. doi: 10.1007/s44154-024-00187-5 (PMC11473752; doi:10.1007/s44154-024-00187-5)
Supplement: Supplementary file 2 — Supplementary Material 2. [file 44154_2024_187_MOESM2_ESM.docx]

Table S1 Cd content and TF of *L. Chuanxiong* in Cd treatment group

| Treatment | Cd content in hydroponic solutions (mg/mL) | Cd content in plant (mg/kg) | Cd content in up-ground part (mg/kg) | Cd content in down-ground part (mg/kg) | TF |
| --- | --- | --- | --- | --- | --- |
| 25 μM Cd | 27.54 | 2969.42 | 275.27 | 2694.14 | 0.102 |
